# Melioidosis, Northeastern Brazil

**DOI:** 10.3201/eid1109.050493

**Published:** 2005-09

**Authors:** Dionne Bezerra Rolim, Dina Cortez Feitosa Lima Vilar, Anastacio Queiroz Sousa, Iracema Sampaio Miralles, Diana Carmen Almeida de Oliveira, Gerry Harnett, Lyn O'Reilly, Kay Howard, Ian Sampson, Timothy J.J. Inglis

**Affiliations:** * Hospital São Jose, Ceará, Brazil;; † Secretaria da Saude do Estado do Ceará, Ceará, Brazil;; ‡ Central Laboratory of Public Health for the State of Ceará, Ceará, Brazil;; § Western Australian Centre for Pathology and Medical Research, Nedlands, Perth, Western Australia, Australia

**Keywords:** Melioidosis, Brazil, Emerging infection, dispatch

## Abstract

Melioidosis was first recognized in northeastern Brazil in 2003. Confirmation of additional cases from the 2003 cluster in Ceará, more recent cases in other districts, environmental isolation of *Burkholderia pseudomallei*, molecular confirmation and typing results, and positive serosurveillance specimens indicate that melioidosis is more widespread in northeastern Brazil than previously thought.

Melioidosis is a fatal bacterial infection found in many parts of the tropical belt, particularly in Southeast Asia and northern Australia. Sporadic cases of the disease have been reported previously in Central and South America (*1*). In 2003 septicemic melioidosis was diagnosed for the first time in northeastern Brazil by culture of the causal agent, *Burkholderia pseudomallei* from a 10-year-old boy (*2*). That case is believed to be the first culture-confirmed case of melioidosis in Brazil and was part of a small cluster of cases (hereafter termed Brazil outbreak 1). At first, evidence that >1 case of melioidosis had occurred was circumstantial. The diagnosis relied entirely on the phenotypic features of a blood culture isolate from the 10-year-old boy. A more detailed, multidisciplinary investigation obtained further evidence for the case cluster and clarified its likely relationship to infection in the surrounding population.

## The Study

**Figure A1:**
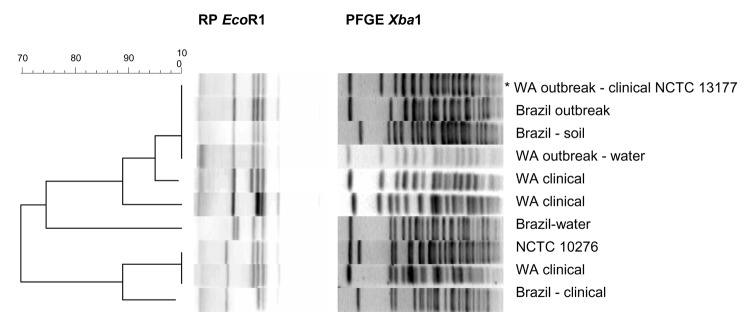
Molecular typing results for *Burkholderia pseudomallei* isolates from Brazil compared with Western Australian and reference isolates. The Unweighted Pair Group Method using arithmetic averages dendrogram on the left refers to the *Eco*R1 ribotype patterns in the center. *Xba*I DNA macrorestriction (pulsed-field gel electrophoresis, PFGE) patterns are shown at the right of the corresponding ribotypes for comparison. The ribotypes and PFGE types are each numbered from the first lane at the top of the figure, increasing by one for each new type. PFGE types were allocated numbers independently of ribotypes.

Outbreak 1 comprised 4 previously healthy children from the Municipality of Tejussuoca; the children were admitted to the hospital with clinical features of systemic infection over the course of 10 days (February 28–March 7, 2003) (Table 1; Figure A1). Three of the children died because of multiple organ systems failure. Patient l died shortly after admission to a local hospital, before any diagnostic microbiology tests could be arranged. Gram-negative bacilli were isolated by blood culture from 2 children, patient 2 and patient 3. For patient 2, the isolate did not survive preliminary laboratory analysis, but findings at autopsy were consistent with melioidosis (*3*). In patient 3, the isolate was presumptively identified as *B. pseudomallei*. Bacterial identification and susceptibility results came too late to guide the treatment of patient 3, who also died, but did lead to changes in antimicrobial drug therapy of patient 4, who was admitted to the hospital later than the other 3 patients, survived, and remains healthy. In her case, melioidosis was demonstrated by laboratory evidence of late seroconversion, detected by indirect hemagglutination assay. Preliminary epidemiologic investigations indicated that the children were probably infected when diving into an irrigation reservoir that filled shortly after the onset of the summer rains.

**Table 1:** Patients implicated in melioidosis case-cluster in northeastern Brazil, 2003 (outbreak 1)

Patient	Sex	Age, y	Outcome	Culture	Autopsy	Serology
1	M	15	Died	NA*	NA	NA
2	F	14	Died	Gram-negative bacteria	Melioidosis	Negative
3	M	10	Died	*Burkholderia pseudomallei*	Melioidosis	Negative
4	F	12	Survived	Negative	NA	Positive

*NA, not available.

Environmental studies conducted shortly after the case cluster occurred (and then repeated with improved methods at a later date) did not isolate *B. pseudomallei* from this location or other nearby sites. A detailed review of surveillance methods was undertaken, and external advice was sought shortly before the ensuing rainy season. The presumptive *B. pseudomallei* isolate from patient 3 was sent to a reference laboratory for independent confirmation and molecular typing. A case definition was established for prospective epidemiologic surveillance, and seroepidemiologic studies began. External advice was sought for environmental isolation methods.

The first clinical isolate (outbreak 1, patient 3, the 10-year-old boy) was confirmed as *B. pseudomallei* by phenotypic and molecular methods, according to a validated discovery pathway (*4*). In brief, polymerase chain reaction (PCR)–based identification, gas-liquid chromatography of fatty acid methyl esters, and an agglutinating monoclonal antibody were used to confirm the isolate presumptively identified as *B. pseudomallei*.

Just over 1 year later, in 2004, several suspected cases of septicemic melioidosis occurred in another location in the State of Ceará (outbreak 2). The *B. pseudomallei* isolate from 1 such patient and 2 *B. pseudomallei* isolates from soil and water samples in the corresponding environmental study were sent for confirmation and molecular typing, as before. The patient was from the Municipality of Banabuiú, ≈400 km from the location of outbreak 1. She used to wash clothing while sitting in a nearby river. She first complained of a perineal abscess, which persisted for 2 weeks before she was admitted to the hospital with septicemia. *B. pseudomallei* was isolated by blood culture after she died. The *B. pseudomallei* environmental isolates were from river water taken near where she washed clothes and from soil from the compacted earth floor under the tub she bathed in at home.

*Eco*R1 ribotyping showed that the *B. pseudomallei* isolate from patient 3 in the 2003 outbreak 1 was similar to the Western Australian outbreak strain (Table 2, Figure A1). *Eco*R1 ribotyping discriminated between 3 of the 4 Brazil *B. pseudomallei* isolates (Brazil outbreak 1, patient 3: ribotype 1; unrelated later case in second district: ribotype 6; and 2 environmental isolates from second district: ribotypes 1 and 4). However, ribotyping was not as discriminating as DNA macrorestriction typing (pulsed-field gel electrophoresis, PFGE), which showed that the Brazil and Western Australian outbreak isolates were distinct strains (Brazil outbreak, patient 3: PFGE type 2, Western Australian outbreak strain: PFGE type 1).

**Table 2:** Molecular typing patterns for Brazil outbreak 1 strain (patient 3), subsequent northeastern Brazilian isolates, and unrelated strains*†

Connection	Location	Type	Source	Culture	Ribotype	PFGE type
Outbreak 1	Ceará, Brazil†	Clinical	Blood	*B. pseudomallei*	1	2
Later case, outbreak 2	Ceará, Brazil†	Clinical	Blood	*B. pseudomallei*	6	9
Later case, outbreak 2	Ceará, Brazil	Environ	Water	*B. pseudomallei*	4	6
Later case, outbreak 2	Ceará, Brazil	Environ	Soil	*B. pseudomallei*	1	3
Cluster	WA, Australia	Clinical	Blood	*B. pseudomallei*	1	1
Cluster	WA, Australia	Environ	Water	*B. pseudomallei*	1	1
Later case 1	WA, Australia	Clinical	Blood	*B. pseudomallei*	2	4
Later case 2	WA, Australia	Clinical	Blood	*B. pseudomallei*	3	5
Later case 3	WA, Australia	Clinical	Blood	*B. pseudomallei*	5	8
NCTC 10276	India	Clinical	Tissue	*B. pseudomallei*	5	7

*PFGE, pulsed-field gel electrophoresis; WA, Western Australia; Environ, environmental; *B. pseudomallei*, *Burkholderia pseudomallei*. †Brazil outbreak 1 occurred at Tejusuoca; outbreak 2 occurred at Banabuiu, both in the state of Ceará.

Autopsy results from patient 2 in the original case cluster were similar to those of the culture-positive third case-patient and were consistent with melioidosis (*3*). The 1 survivor of the case cluster (Brazil outbreak 1, patient 4) seroconverted (titer = 1:160) from an undetectable indirect hemagglutination assay (IHA) titer around the time of her infection. One parent was borderline positive by IHA (titer of 1:40), and one was negative by IHA (<10).

Of the 36 persons from both districts tested by IHA for serologic evidence of exposure to *B. pseudomallei*, 14 had titers >1:40; 7 had a titer of >1:160; and 2 had titers of 1:5,120. No significant associations occurred between seropositivity and district, or seropositivity and age. However, seropositivity and sex were significantly associated (Fisher exact test, p = 0.0159, relative risk = 0.320, 95% confidence interval [CI] = 0.12–0.83); 10 of 16 female patients had titers >40, the threshold titer, compared to 4 of 20 male patients. This apparent association between female sex and seropositivity is the reverse of the association expected from experience in Southeast Asia.

## Conclusions

At the time of writing, sporadic human infection has been reported sporadically from other locations in northeastern Brazil, consistent with an emerging infectious disease. Prospective case surveillance, improved laboratory diagnosis, and targeted environmental bacteriologic testing will help clarify the epidemiology of melioidosis in this region. Why this disease has appeared in Brazil remains obscure, although our preliminary molecular typing results indicate a possible link with Australian and Southeast Asian infections through a putative common progenitor strain. Veterinary investigation may help identify a possible means of introduction of the disease, since the goats that feature in subsistence farming in Ceará may have been imported from parts of the Caribbean where caprine melioidosis is known to occur (*5*). An alternative hypothesis is that *B. pseudomallei* was introduced through rice cultivation. An environmental search for *B. pseudomallei* (then *Pseudomonas pseudomallei*) was conducted shortly after melioidosis was first reported in South and Central America (*6*): a large number of water samples from rice fields near São Paulo were cultured. No *P. pseudomallei* was found, which led to the conclusion that the temperature and moisture of the environment did not favor the microorganism. More recently, *B. pseudomallei* has been presumptively identified in the root soil of sugar cane in the São Paulo region (*7*). Rice is grown in parts of Ceará where melioidosis cases have been identified and is an important crop in other parts of the country. Further epidemiologic and environmental studies are needed to determine the extent of the environmental hazard and the risk it represents for the human population and their livestock in northern Brazil. Finally, the terrain in Ceará has many similarities to northern Australia, where the summer rains are known to coincide with most septicemic melioidosis cases (*8*). In some parts of the Caribbean, sporadic melioidosis cases appear to be a harbinger of more common disease when flooding or other climatic determinants prevail (*9**,**10*). The surveillance methods recently introduced in Ceará may therefore help predict future melioidosis events. Data on melioidosis serology results in an epidemic setting are limited. The seroepidemiology survey conducted after the Western Australian melioidosis outbreak relied on access to previous serum samples from the same community, fortuitously collected for other purposes before the outbreak (*11*). Results from a serologic survey based on single samples from each study participant, as in Ceará, will necessarily have much wider CI. The investigation into the Western Australian outbreak identified persons who seroconverted without clinical evidence of *B. pseudomallei* infection. Carefully planned prospective seroepidemiologic studies in northeastern Brazil will clarify the importance of these preliminary observations. Establishing the true prevalence of melioidosis in northeastern Brazil will help ascertain the threshold for serodiagnosis and the clinical relevance of borderline results.

In the absence of any obvious anthropogenic changes known to increase melioidosis risk, the unusual weather systems operating in early 2003 appear to be the most likely explanation for the apparent temporal and positional clustering of cases. The diversity of molecular types of *B. pseudomallei* and the seroprevalence of *B. pseudomallei* antibody–positive persons are more consistent with an endemic disease that has gone undetected for several years than a recent, point-source incursion. Further epidemiologic studies will need to address whether the apparent emergence of melioidosis in northeastern Brazil is due primarily to improved ascertainment, the regional impact of climate change, changes in land use, or a combination of these factors.
